# Effect of Instruction on Preventing Delayed Bleeding after Colorectal Polypectomy and Endoscopic Mucosal Resection

**DOI:** 10.3390/jcm10050928

**Published:** 2021-03-01

**Authors:** Takuya Okugawa, Tadayuki Oshima, Keisuke Nakai, Hirotsugu Eda, Akio Tamura, Ken Hara, Tomohiro Ogawa, Tomoaki Kono, Takashi Kondo, Katsuyuki Tozawa, Masashi Fukushima, Toshihiko Tomita, Hirokazu Fukui, Jiro Watari, Hiroto Miwa

**Affiliations:** Division of Gastroenterology, Department of Internal Medicine, Hyogo College of Medicine, Nishinomiya 663-8501, Japan; okugawat@hyo-med.ac.jp (T.O.); k-nakai@hyo-med.ac.jp (K.N.); eda@hyo-med.ac.jp (H.E.); akio@hyo-med.ac.jp (A.T.); k-hara@hyo-med.ac.jp (K.H.); to-ogawa@hyo-med.ac.jp (T.O.); kono@hyo-med.ac.jp (T.K.); kondou@hyo-med.ac.jp (T.K.); katu-you@hyo-med.ac.jp (K.T.); ma-fukushima@hyo-med.ac.jp (M.F.); tomita@hyo-med.ac.jp (T.T.); hfukui@hyo-med.ac.jp (H.F.); watarij@kinentou.or.jp (J.W.); miwahgi@hyo-med.ac.jp (H.M.)

**Keywords:** colorectal polyps, delayed bleeding, antithrombotic agents, cold snare polypectomy, instruction

## Abstract

Background: The frequency of delayed bleeding after colorectal polypectomy has been reported as 0.6–2.8%. With the increasing performance of polypectomy under continuous use of antithrombotic agents, care is required regarding delayed post-polypectomy bleeding (DPPB). Better instruction to educate endoscopists is therefore needed. We aimed to evaluate the effect of instruction and factors associated with delayed bleeding after endoscopic colorectal polyp resection. Methods: This single-center, retrospective study was performed to assess instruction in checking complete hemostasis and risk factors for onset of DPPB. The incidence of delayed bleeding, comorbidities, and medications were evaluated from medical records. Characteristics of historical control patients and patients after instruction were compared. Results: A total of 3318 polyps in 1002 patients were evaluated. The control group comprised 1479 polyps in 458 patients and the after-instruction group comprised 1839 polyps in 544 patients. DPPB occurred in 1.1% of polyps in control, and 0.4% in after-instruction. Instruction significantly decreased delayed bleeding, particularly in cases with antithrombotic agents. Hot polypectomy, clip placement, and use of antithrombotic agents were significant independent risk factors for DPPB even after instruction. Conclusion: The rate of delayed bleeding significantly decreased after instruction to check for complete hemostasis. Even after instruction, delayed bleeding can still occur in cases with antithrombotic agents or hot polypectomy.

## 1. Introduction

Endoscopic resection is an effective treatment for colorectal polyps to achieve en bloc resection and prevent local recurrence or the development of cancers and mortality [1,2,3,4]. Indications for endoscopic treatment have broadened, even in high-risk conditions such as patients still receiving antithrombotic agents, which are seeing increasing use [5]. However, delayed post-polypectomy bleeding (DPPB) remains one of the major adverse events. The incidence of DPPB has not decreased in recent years, due to the aging of society and the increased use of antithrombotic agents [6]. Mortality directly attributable to DPPB is scarce, but this event can be serious because of unpredictable onset often occurring after hospital discharge and requiring intensive management involving repeated colonoscopy, blood transfusion, hospitalization, and a few colectomies [7].

Previous studies have reported risk factors for DPPB of age, size, location, chronic kidney disease, liver cirrhosis, and use of antithrombotic agents [6,8,9]. Patients receiving antithrombotic agents are more prone to experience DPPB than those not taking these agents [6]. Management of antithrombotic use in patients undergoing polypectomy is an important issue in clinical practice. Cessation of antithrombotic agents before treatment is the easiest option, but increases the risk of thrombosis [10,11,12]. Clarifying what options are available to decrease the risk of DPPB after endoscopic polypectomy is therefore important.

Recently, cold snare polypectomy (CSP) has been reported as a safe procedure even in patients on antithrombotic medications [7,13,14]. Although some reports have found no DPPB after CSP, this event still occurs under real-world clinical conditions, and whether placement of clips is effective for preventing DPPB remains controversial [15,16,17,18]. Furthermore, no reports have clarified whether DPPB can be prevented by checking for complete hemostasis after polypectomy, or by inspection of the mucosal defect in the resection stump after resection and post-resection irrigation after CSP [19,20], although both steps are considered effective. These kinds of basic procedures are likely important for the prevention of DPPB after endoscopic resection. Accordingly, the present study was conducted to evaluate the effects of instruction in checking for complete hemostasis and to identify factors associated with colorectal DPPB.

## 2. Methods

### 2.1. Study Design, Setting and Participants

We performed this retrospective cohort study in a single center to evaluate the effects of instruction in checking for complete hemostasis after colorectal polyp resection on DPPB rates. All cases of polypectomy and endoscopic mucosal resection were included in this study at Hyogo College of Medicine between January and December 2018. Written informed consent was obtained from all patients prior to endoscopic procedures. The study protocol conforms to the ethical guidelines of the World Medical Association Declaration of Helsinki as reflected in a prior approval by the institution’s human research committee. The study was approved by the Ethics Review Board, Hyogo College of Medicine, Japan, on 8 May 2020 (No. 2413). The database of colonoscopies with polyp resection and medical records of patients were evaluated with respect to the patient- and polyp-related factors noted below.

### 2.2. Interventions

**Figure 1:**
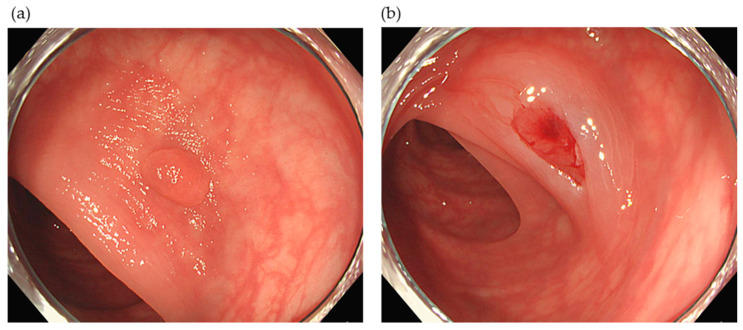
Representative pictures of cold snare polypectomy. (**a**) A 5-mm polyp in the transverse colon. (**b**) The mucosal defect is expanded by water irrigation and no bleeding is evident.

As the incidence of DPPB was thought to be high in the first half of 2018, pictures and preserved videos were assessed when DPPB was identified. Inspection while taking pictures of mucosal defects after polypectomy had not been routinely performed in cases with DPPB, particularly after cold polypectomy. Doctors performing polypectomy were therefore clinically instructed to confirm complete hemostasis on 18 June 2018. The detailed instruction (Table 1) was to confirm hemostasis by inspecting and taking pictures of the mucosal defect at the resection stump after all types of polypectomies. Washing the mucosal defect with water was recommended after all types of polypectomies and irrigation with water to achieve pseudo-submucosal injection was recommended after CSP (Figure 1). The addition of hemostatic clip placement was recommended when bleeding did not stop for more than 30 s after all types of polypectomies.

### 2.3. Primary Outcome

The primary outcome of this study was predefined as the effect of instruction on the frequency of DPPB. We also sub-analyzed the influence of instruction on treatment strategies and residual risk factors even after instruction. Patients were divided into two groups: historical control and after-instruction groups. The characteristics of patients and polyps before and after instruction were then compared. Cases with endoscopic submucosal dissection (ESD) and patients with familial adenomatous polyps (FAP) were excluded.

### 2.4. Polyp Resection Procedure

Colonoscopic polypectomy was performed using a normal electric video endoscope (PCF-H290ZI, PCF-Q260AZI, CF-HQ290; Olympus, Tokyo, Japan), with carbon dioxide insufflation. All patients received 2 L of polyethylene glycol solution or 1.8–2.4 L of magnesium citrate solution in the morning on the day of the procedure. CSP was performed for small polyps (≤10 mm in maximum diameter) using Captivator II (Boston Scientific Japan, Tokyo, Japan). Cold forceps polypectomy (CFP) was performed for small polyps (≤4 mm in maximum diameter) using Radial Jaw4 Jumbo cold polypectomy forceps (Boston Scientific Japan, Tokyo, Japan). The same endoscopists that performed CSP used a B-wave (Xemex, Tokyo, Japan), Captivator II (Boston Scientific Japan, Tokyo, Japan), or SnareMaster (Boston Scientific Japan, Tokyo, Japan) to perform hot snare polypectomy (HSP). For endoscopic mucosal resection (EMR), normal saline was injected into the submucosa before excision. VIO 300D (ERBE Elektromedizin, Tubingen, Germany) was used as a power source, and all participant endoscopists used the same setting. Decisions on procedures for polyps 3–45 mm in size (i.e., application of HSP, EMR, CSP or CFP) and decisions regarding clip placement for prophylaxis of PPB were made by the endoscopists in charge.

### 2.5. Factors for Assessment

DPPB was defined as overt rectal bleeding requiring endoscopic hemostasis and occurring between 24 h and 28 days after the procedure [13,21]. When adhesion of blood clot or active bleeding was detected at mucosal defects, they were considered as bleeding sites. When we could not decide which possible mucosal defects with blood clot were the origin of bleeding in a patient, multiple sites were considered as the origin. Risk factors were identified by comparisons between historical control and after-instruction groups. Risk factors for DPPB were classified according to patient, polyp, and procedure. Patient-related factors consisted of age, sex, comorbidities (cerebrovascular disease, diabetes mellitus, hemodialysis, hypertension, and ischemic heart disease), number of polyps removed per patient, and use of antithrombotic agents (anticoagulants including warfarin and direct oral anticoagulants, antiplatelet drugs including aspirin, clopidogrel, and cilostazol). If medications were only used intermittently, the patient was not classified as a user. Antithrombotic agents were discontinued or changed based on guidelines established by the Japanese Gastroenterological Endoscopy Society [22,23] throughout the study period. When antithrombotic agents were discontinued or changed based on the guidelines, the patient was still defined as taking antithrombotic agents. Polyp-related factors comprised size, gross morphology, and location of the polyp. Polyp size was measured in comparison to the resection devices and divided into 3 groups (<6 mm; 6–10 mm; or ≥11 mm). The gross morphologies of polyps were classified as either pedunculated or sessile type, according to the Paris classification [24]. Locations of polyps were defined as right-sided colon (cecum, ascending colon, and transverse colon) or left-sided colon (descending colon, sigmoid colon, and rectum). Procedure-related factors included resection methods (COLD: CSP and CFP; or HOT: HSP and EMR) and use of prophylactic clipping and hemostatic clipping. Endoscopist experience (≥10 years) was also assessed between groups.

### 2.6. Statistical Analysis

No data regarding preventive effects on DPPB with instruction were available. However, a 1% decrease in DPPB from the reported incidence (1.4%) [25] was estimated. Based on these criteria, 1399 polyps in each group were required to detect a 5% (two-sided) intergroup difference in the primary variable using the chi-square test. Considering a dropout rate of approximately 5%, we set a required total of 2940 polyps (1470 per group).

Patient-related, polyp-related, and procedure-related characteristics were compared using chi-square testing or Fisher’s exact tests, and Mann-Whitney U tests, where appropriate. To determine risk factors associated with DPPB, we estimated the odds ratio (OR) and 95% confidence interval (CI). Univariate analysis and forward stepwise (likelihood ratio) logistic regression analysis were performed to test the influence of several factors in association with DPPB. All reported *p*-values were two-sided and values less than 0.05 were considered statistically significant. SPSS 22.0 (SPSS Inc., Chicago, IL, USA) was used for all statistical analyses.

## 3. Results

### 3.1. Characteristics of Patients

**Figure 2:**
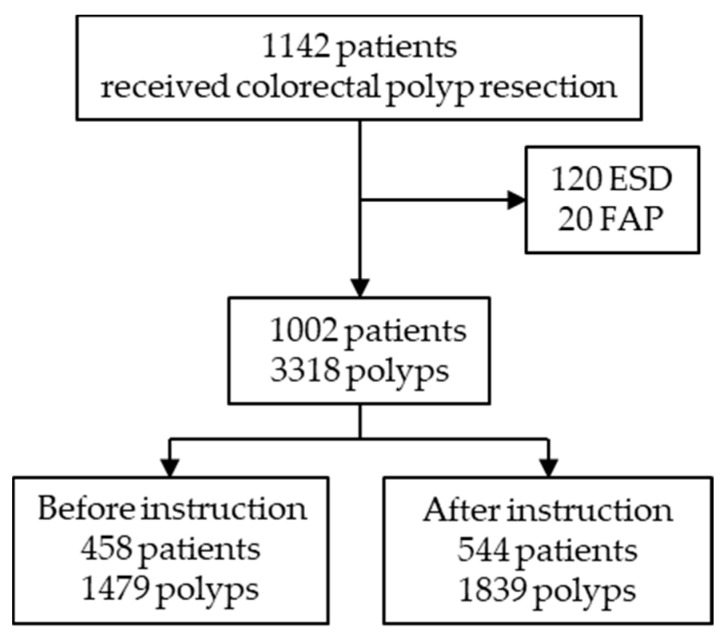
Flow diagram of patients and polyps. ESD: endoscopic mucosal dissection; FAP: familial adenomatous polyposis.

A total of 1142 patients received colorectal polyp resection between January and December 2018. Of those, 120 cases with ESD and 20 patients with FAP were excluded. A total of 3318 polyps in 1002 patients were evaluated and assigned to the two groups: 1479 polyps in 458 historical control patients, and 1839 polyps in 544 patients after instruction (Figure 2). After instruction, the rate of taking pictures of mucosal defects after polypectomy increased from 71.6% to 93.6% on a per-polyp basis. Table 2 shows the background characteristics of control patients and patients after instruction. Among all cases, 207 patients (20.7%) with 784 polyps (23.6%) received antithrombotic agents. No significant differences between groups were seen regarding clinical features including age, sex, or baseline characteristics including use of antithrombotic agents, and number of polyps removed per patient. The incidence of background comorbidities did not differ in control and after-instruction groups. Forty-seven endoscopists performed the procedures (13 endoscopists with experience more than 10 years, 34 endoscopists with experience less than 10 years). The experience of the endoscopist (≥10 years) in charge of polypectomy was significantly higher after instruction (control, 31.0% vs. instruction, 38.6%; *p* < 0.05). This difference was not planned in the original instruction.

### 3.2. Characteristics of Procedure

The following assessments were all performed on a per-polyp basis. The characteristics of polyps and procedures were compared between control and after-instruction groups. Polyp size, pedunculated type, and polyp location did not differ between control and after-instruction groups (Table 3). The rate of COLD was not significantly different between the two groups.

### 3.3. Incidence of DPPB

Among the total cohort, DPPB occurred in 20 patients (2.0%) and 24 polyps (0.7%). Two patients with two possible defects in control and one patient with three possible defects in after-instruction were counted. Endoscopic hemostasis was successful in all cases of DPPB. Clips had been placed in 14 polyps (58.3%) before DPPB. DPPB occurred at a median of 1 day (IQR, 1–3 days) after treatment. The rate of DPPB was significantly lower in after-instruction (0.4%) compared to control (1.1%; *p* < 0.05) (Table 4). When considering the method of polypectomy, both COLD and HOT groups showed lower rates of DPPB after instruction, and the rate of DPPB in the COLD group was significantly lower after instruction (0.4% vs. 0%; *p* < 0.05). In the COLD group, the incidence of DPPB was 0.2% in total and 0.4% in control. Three of four polyps with DPPB were in patients using antithrombotic agents in control, and no DPPB occurred in after-instruction. In the HOT group, the incidence of DPPB was 1.6% in total and tended to decrease after instruction. Additionally, when considering the use of antithrombotic agents, the rate of DPPB with antithrombotic agents was significantly lower after instruction (2.6% vs. 0.5%, *p* < 0.05). On the other hand, the rate of DPPB without antithrombotic agents did not differ between control and after-instruction groups.

### 3.4. Factors Related to DPPB

All resected polyps were divided into bleeding and non-bleeding groups. Uni- and multivariate logistic regression analyses were performed to identify factors related to DPPB (Table 5). Univariate analysis showed that instruction was a significant preventive factor for DPPB. Furthermore, polyp size, pedunculated type, HOT, clip placement, and use of antithrombotic agents were significant risk factors for DPPB. No significant differences in location (right-side colon) or endoscopist experience (≥10 years) were seen between bleeding and non-bleeding groups. Multivariate logistic regression analysis showed that instruction was a significant independent preventive factor for DPPB. HOT, clip placement, and use of antithrombotic agents were significant independent risk factors for DPPB.

## 4. Discussion

This retrospective cohort study showed the importance of checking to ensure complete hemostasis by inspecting mucosal defects of the resection stump. Even after instruction, a small number of DPPB events still occurred after HSP. As clipping was an independent risk factor for DPPB, the location of clipping should be further considered to decrease DPPB. To the best of our knowledge, the present study is the first to show the importance of looking at the mucosal defect after polypectomy. Although the procedure time due to inspection of mucosal defects after polypectomy must have been prolonged, any DPPB that arises risks becoming serious because of unpredictable onset often occurring after hospital discharge and requiring intensive management [7]. In a recent meta-analysis of DPPB, the frequency was reported as 0.6–2.8% [26]. Cardiovascular disease, hypertension, polyp size (>10 mm), and polyps located in the right colon were indicated as significant risk factors for DPPB, whereas age, sex, alcohol use, smoking, diabetes, cerebrovascular disease, pedunculated morphology, and carcinoma histology were not significant risk factors for DPPB [26]. The use of antithrombotic agents is one of the risk factors for PPB, and both immediate and delayed PPB occur in patients taking antithrombotic agents. In the present study, interestingly, the rate of DPPB in patients taking antithrombotic agents decreased significantly after instruction. However, the use of antithrombotic agents was still a significant risk factor for DPPB. These data indicate that inspection of the mucosal defect after polypectomy may be effective for patients taking antithrombotic agents, though it is important to note that the inspection cannot fully mitigate the risk of DPPB for these patients.

The experience of the endoscopist might also be associated with the incidence of DPPB. Less than 300 procedures or less than one year of experience were reported to correlate with higher rates of DPPB [27,28]. Conversely, Kwon et al. did not find any association in DPPB rate comparing endoscopists practicing more or less than 10 years [29]. Similarly, Lee et al. found no difference in DPPB rate between procedures performed by fellows versus staff [30]. These data were similar to the present study, in that endoscopist experience did not affect the rate of DPPB. Therefore, constant improvement and training are needed that should not only be aimed at residents and novice practitioners, but also experienced doctors. As a unified definition of endoscopic experience was lacking from these studies, whether endoscopic experience affects the incidence of DPPB remains unclear and further studies are warranted.

In a meta-analysis of hot and cold polypectomy, the rates of bleeding were 0.8% and 0% on a per-patient basis, and 0.4% vs. 0% on a per-polyp basis [31]. As indicated before, CSP is safer than HSP. In addition, in the present study, HOT was a significant risk factor for DPPB in multivariate analysis. As the incidence of bleeding was small, we need to be careful before concluding that the method of polypectomy affects the DPPB. However, the procedure itself must be one of the important factors contributing to the incidence of DPPB.

The incidence of clipping after cold polypectomy was 0–6% in previous reports [21,32,33,34,35]. Interestingly, all prospective data showed no DPPB after cold polypectomy, suggesting the safety of CSP. However, the incidence of DPPB after cold polypectomy in the present study could not be ignored before the intervention. The data indicated that we still need to be careful about DPPB even after cold polypectomy, with precise inspection of the mucosal defect. In the present study, no DPPB was seen after instruction in polyps after CSP, even with the increased rate of the procedure. As immediate PPB was higher after CSP compared to HSP in a meta-analysis [36], we also need to be careful regarding DPPB. However, when irrigation with water to make a pseudo-submucosal injection is done and we can confirm hemostasis after CSP by inspection of the mucosal defect after polypectomy, the situation can be considered safe and no DPPB occurs.

Although the rate of clipping increased after instruction in expectation of preventing DPPB in the present study, clipping was indeed an independent risk factor for DPPB. This was unexpected, but as recent reports have indicated, clipping does not appear effective in preventing DPPB [15]. One report showed that clipping after cold polypectomy was more likely to be used in the antithrombotic group. However, no significant difference in the rate of DPPB was seen between lesions with and without clipping in that study [7]. One explanation might be related to the target lesions for clipping. Indications for clipping in the present study depended on the endoscopist in charge. When the risk of DPPB is considered high, clipping may be added. Therefore, lesions with clipping might already be at higher risk of DPPB, with clipping not proving effective for preventing DPPB. Another contributor would be ineffective clipping. The location of clipping should be reconsidered for preventing DPPB, because DPPB sometimes occurred next to the residual clips. Further studies are warranted to evaluate the effects of clipping on prevention of DPPB with careful inspection of mucosal defects.

Other than CSP and clipping, no other interventions have been assessed for the prevention of DPPB. In the present study, instruction on inspection of the mucosal defect proved preventive for DPPB, and no DPPB was seen after CSP even with continued use of antithrombotic agents. These data indicate that the intervention presented here would likely already have been enacted in clinical studies assessing the safety and efficacy of CSP, because they tried to avoid DPPB after CSP. Interestingly, no reports have shown the efficacy of clipping after CSP. A study by Kawamura et al. that was not permitted to add prophylactic hemostatic clipping after CSP for 5- to 9-mm polyps did not show any DPPB in 346 polyps [21]. The safety and efficacy of CSP can thus be achieved under strict inspection of mucosal defects with injection of water and without prophylactic clipping [21,32].

Some limitations to the present study must be considered, and may require attention in further investigations. First, this was a retrospective study from a single institution. We therefore cannot exclude unrevealed confounding factors that affect DPPB. As our institution is a tertiary care center, the doctors would have encountered polypectomy procedures in different institutes earlier in their careers, and may have had different backgrounds and be likely to show the data of a multicenter study. Although performing a randomized study to examine the effects of inspection of mucosal defects may encounter ethical problems, prospective studies that assess the effect of irrigation of the mucosal defect after CSP might be possible. A second limitation was that the frequency of bleeding was low. As equipment for treatment differs between institutes and is improving quickly, a multicenter prospective study of a larger cohort would provide stronger evidence and reduce the risk of Berkson’s bias.

## 5. Conclusions

The present study indicated that the rate of DPPB significantly decreased after instruction to check for complete hemostasis. Even after instruction was given, delayed bleeding was still encountered in cases with antithrombotic agents. Efforts to minimize events that may cause serious problems are still needed.

## Figures and Tables

**Table 1:** Instructions.

To confirm hemostasis by inspecting and taking pictures of the mucosal defect at the resection stump after all types of polypectomies To wash the mucosal defect with water after all types of polypectomies To water irrigate to achieve pseudo-submucosal injection after cold snare polypectomy To add hemostatic clip placement when bleeding does not stop for more than 30 s after all types of polypectomies

**Table 2:** Characteristics of patients.

	Control (*n* = 458)	Instruction (*n* = 544)	*p* Value
Age, y	70 (61–77)	69 (61–77)	0.88
Sex, male	299 (65.3)	362 (66.5)	0.69
Antithrombotic agents	94 (20.5)	113 (20.8)	0.94
Anticoagulation agents	21 (4.6)	31 (5.7)	0.43
Antiplatelet agents	78 (17.0)	91 (16.7)	0.72
Antiplatelet and anticoagulation	5 (1.1)	9 (1.7)	0.58
NSAIDs	11 (2.4)	20 (3.7)	0.28
Cerebrovascular disease	20 (4.4)	24 (4.4)	1
Diabetes mellitus	102 (22.3)	99 (18.2)	0.11
Hemodialysis	9 (2.0)	16 (2.9)	0.42
Hypertension	213 (46.5)	236 (43.4)	0.34
Ischemic heart disease	14 (3.1)	13 (2.4)	0.56
Number of polyps removed per patient	2 (1–4)	2 (1–4)	0.87

y: years. Control: historical control group. Instruction: after-instruction group. Values are median (interquartile range) or *n* (%).

**Table 3:** Characteristics of polyps and procedures.

		Control (*n* = 1479)	Instruction (*n* = 1839)	*p* Value
Size	≤5 mm	814 (55.0)	1074 (58.4)	0.09
	6–10 mm	579 (39.1)	652 (35.5)	
	≥11 mm	86 (5.8)	113 (6.1)	
Morphology; Ip		55 (3.7)	62 (3.4)	0.63
Location; right-sided colon		891 (60.2)	1111 (60.4)	0.94
Procedure; COLD		944 (63.8)	1132 (61.6)	0.18
Clip		144 (9.7)	416 (22.6)	<0.01

Control: historical control group. Instruction: after-instruction group. Values are *n* (%).

**Table 4:** Incidence of delayed post-polypectomy bleeding.

		Control	Instruction	*p* Value
All (*n* = 3318)		17/1479 (1.1)	7/1839 (0.4)	<0.05
Procedure	COLD (*n* = 2076)	4/944 (0.4)	0/1132 (0)	<0.05
	HOT (*n* = 1242)	13/535 (2.4)	7/707 (1.0)	0.07
Antithrombotic agents	+ (*n* = 784)	10/383 (2.6)	2/401 (0.5)	<0.05
	− (*n* = 2534)	7/1096 (0.6)	5/1438 (0.3)	0.38

Control: historical control group. Instruction: after-instruction group. Values are *n* (%).

**Table 5:** Factors related to delayed post-polypectomy bleeding.

		Univariate OR (95% CI)	*p* Value	Multivariate ^*^ OR (95% CI)	*p* Value
Intervention	Control	1.0		1.0	
	Instruction	0.33 (0.13–0.80)	<0.05	0.21 (0.08–0.53)	<0.01
Size	≤5 mm	1.0			
	6–10 mm	5.42 (1.78–16.5)	<0.01		
	≥11 mm	14.6 (4.10–52.3)	<0.01		
Morphology	non-Ip	1.0			
	Ip	7.46 (2.14–21.1)	<0.01		
Location	Left-sided colon	1.0			
	Right-sided colon	1.31 (0.53–3.57)	0.68		
Procedure	COLD	1.0		1.0	
	HOT	8.47 (2.83–34.1)	<0.01	3.66 (1.02–13.2)	<0.05
Clip	(−)	1.0		1.0	
	(+)	7.04 (2.89–17.8)	<0.01	4.86 (1.90–12.4)	<0.01
Experience of endoscopist	<10 y	1.0			
	≥10 y	0.80 (0.33–2.02)	0.67		
Antithrombotic agents	(−)	1.0		1.0	
	(+)	3.26 (1.33–7.98)	<0.01	4.45 (1.74–11.4)	<0.01

CI: confidence interval. OR: odds ratio. y: years. Control: historical control group. Instruction: after-instruction group. * Age and sex adjusted forward stepwise (likelihood ratio) logistic regression analysis.

## Data Availability

The data presented in this study are available on request from the corresponding author. The data are not publicly available due to ethical restrictions.
